# Efficacy of pre-emptive use of cyclooxyenase-2 inhibitors for total knee arthroplasty: a mini-review

**DOI:** 10.1186/s42836-019-0015-3

**Published:** 2019-11-27

**Authors:** Jianda Xu, Huan Li, Chong Zheng, Bin Wang, Pengfei Shen, Zikang Xie, Yuxing Qu

**Affiliations:** 1 Department of Orthopaedics, Changzhou Traditional Chinese Medical Hospital, Affiliated Hospital of Nanjing University of Traditional Chinese Medicine, 25 North Heping Road, Changzhou, 213000 Jiangsu China; 2 Department of bone and joint, The First People’s Hospital of Changzhou, The Third Affiliated Hospital of Suzhou University, Changzhou, 213003 China

**Keywords:** Cyclooxyenase-2 inhibitors, Pre-emptive analgesia, Total knee arthroplasty

## Abstract

Total knee arthroplasty (TKA) is regarded as the most effective surgery for patients with later-stage arthritis of the knee, but the postoperative pain management for functional improvement of the knew is still a challenging task. This review discusses the mechanism by which the selective cyclooxyenase-2 inhibitors, which reduce the peripheral and central sensitization, decrease pain after TKA. This review also covers the protocols, safety, efficacy, and progress of cyclooxyenase-2 inhibitors in pre-emptive analgesia.

## Introduction

TKA is believed to be the most effective surgery for patients with later-stage arthritis of the knee. Postoperative pain management is a key to better rehabilitation and more favorable clinical outcomes [1, 2]. Although a large number of analgesic drugs and methods are being used, the postoperative pain management is still a challenging task. The preemptive analgesia has been proved to be an effective method for relieving postoperative pain. And the selective cyclooxyenase-2 inhibitors are regarded as an important drug for preemptive analgesia. This paper briefly reviews the application of the selective cyclooxyenase-2 inhibitors in reducing the peripheral and central sensitization, and discussess the related protocols, safety, efficacy of the agents in pre-emptive analgesia.

## Pain management in total knee arthroplasty (TKA)

Many analgesic innovations (including intravenous patient-controlled analgesia, peripheral nerve blockade and epidural analgesic technique) have been used to reduce the pain level post-operatively, but none of them is proved an optimum choice, such as an enduring effect of opium. Multimodal analgesia is a preferable approach for relieving postoperative pain with minimal side effects [3]. Preemptive analgesia initiated before surgery to decrease pain in the early postoperative period is more effective than the similar analgesic techniques initiated after surgery. This strategy reduces the postoperative abnormal sensitivity of peripheral and central neurons, which prolonge pharmacological duration and decrease the level of postoperative pain [4].

## Preemptive analgesia

The preemptive analgesia is not a fresh idea. It was proposed in the early twentieth century. It has been proved to be an effective method for relieving postoperative pain [5]. Armitage [6] suggested that pain prevention is better than pain relief. Gottchalk et al. [7] suggested that preemptive epidural analgesia significantly attenuated postoperative pain during hospitalization and even after discharge, compared with aggressive pain management. The pain after TKA is a kind of sharp pain. Many analgesics and analgesic interventions have been available, including nonsteroidal anti-inflammatory drugs (NSAIDs), opioids, N-methyl-D-aspartate receptor antagonists, peripheral local anesthetics, systemic antiepileptics, etc. [8–12]. Unlike opioids, NSAIDs have antipyretic actions but with less side effects [13]. Therefore, NSAIDs are usually prescribed as alternatives to or adjuncts of opioid-based analgesia.

## NSAIDs inhibits cyclooxyenase-2 (COX-2)

The mechanism of action of NSAIDs is inhibition of arachidonic acid COX pathways [14]. The nonselective NSAIDs inhibit COX-2 enzymes. There are two isoforms of COX enzymes, i.e., COX-1 and COX-2 [15]. COX-1 is the normally expressed and plays roles in gastric protection, platelet aggregation, and renal blood flow maintenance. Inhibiting COX-1 enzyme results in dysfunction of platelets and gastrointestinal toxicity. COX-2 expresses minimally in the normal settings, and its expression was markedly up-regulated following traumas and surgeries [16]. Therefore, the selective COX-2 inhibitors are used for perioperative pain management [17]. In patients who underwent TKA, Xu et al. [18] found the protocol of preemptive analgesia with multimodal analgesics added improved analgesic effect, reduced inflammatory reaction, and accelerated functional recovery in the first postoperative week. However, it didn't help in improving the long-term function of the knee.

## NSAIDs inhibits peripheral and central sensitization

The analgesic effects of NSAIDs include peripheral and spinal ones. Noxious stimuli are stimuli that elicit tissue damages and activate nociceptors. Nociceptors are sensory receptors that pick up the signals from the damaged tissues. An increased sensitivity and decreased stimulus threshold tend to occur after injury, both contributing to hypersensitivity to pain [19]. NSAIDs inhibit peripheral sensitization of the primary afferent nerve terminals by reducing the persistent afferent barrage. The latter also contributes to the sensitization of the central neurons [20].

Prostaglandins are primary noxious mediators induced by noxious stimuli. Prostaglandins activate the primary afferents and induce sensitization of nociceptors. Prostaglandins sensitize receptors at the injury site, involving peripheral and spinal neurosystems. The peripheral effects elicit the peripheral anti-inflammatory actions by inhibiting the synthesis of prostaglandins through the inactivation of COXs. Prostaglandins only sensitize the receptors but do not directly produce pain. Both isoforms of COX enzymes regulate the synthesis of prostaglandins. However, prostaglandins synthesized by COX-2 usually cause pain and inflammatory reaction. If the COX-2 pathway is selectively interrupted, pain will be reduced significantly with less adverse events. That means the primary mediators are blocked and the activation of second downstream mediators are inhibited significantly. Considering the expression of COX-2 in glial cells and dorsal horn neurons, selective inhibition of COX-2 also minimizes the central sensitization. The connection and cellular metabolism styles in the dorsal horn are altered [21]. Therefore, the preemptive use of selective COX-2 inhibitors can be an effective supplement to multimodal analgesia in relieving postoperative pain.

In this regard, NSAIDs are more accurate anti-hyperalgesics than analgesics in terms of action. Upon stimulation by noxious stimuli, the damaged cell membranes release arachidonic acid that is converted into prostaglandin E2 (PGE2) by COX-2. PGE2 plays a critical role in nociceptor activation and initiation of the inflammatory cascade. On the basis of this reaction, NSAIDs decrease inflammatory hyperalgesia and raise the pain threshold. NSAIDs also block the recruitment of leukocytes and monocytes, and the production of cytokines, and other leukocyte-derived inflammatory mediators [22, 23]. The spinal effects are responsible for a consequent reduction in spinal NMDA-mediated reactions in the spinal cord [24]. In addition to the central mechanism, NSAIDs can also inhibit spinal prostanoid synthesis by reducing release of neurotransmitters from pain reflex arc [25]. Some NSAIDs are able to cross the blood-brain barrier to reach brain, where they limit PGE2 synthesis in sensitized neurons and glial cells [26]. Therefore, NSAIDs can decrease local inflammatory cascade, as well as peripheral and central sensitization.

## COX-2 inhibitors in preemptive analgesia

A meta-analysis study showed that a single dose of either etoricoxib or celecoxib is effective for postoperative pain relief [27, 28]. Therefore, this selective inhibition of COX-2 becomes the main choice for preemptive analgesia with less side effects on gastrointestinal function and platelet aggregation, especially, in orthopaedic surgeries. Kashefi et al. [29] reported that preemptive use of 200 mg oral celecoxib 2 h before surgery significantly reduced pain intensity till 4 h after surgery compared with placebo in patients who underwent lower extremity orthopaedic surgeries. In arthroscopic knee surgeries, preoperative administration of rofecoxib is a successful method to control acute pain postoperatively [30]. Reuben et al. [31] evaluated the safety and efficacy of rofecoxib used before TKA. They suggested that rofecoxib could significantly decrease postoperative pain, but did not increase 24-h blood loss or transfusion rate. Xu et al. [19] found that in patients who underwent TKA, preemptive analgesia added to multimodal analgesic regime improved analgesia, reduced inflammatory reaction, and accelerated functional recovery in the first postoperative week. However, it did not improve the long-term function. The selective COX-2 inhibitors were initially used as a means to relieve chronic pain, and demonstrated a anti-pain efficacy similar to NSAIDs. Currently, the selective COX-2 inhibitors are the main analgesic agents that can be used immediately after surgery. Buvanendran et al. [32] found that perioperative use of selective COX-2 inhibitors could ease acute pain and improve flexion of the knee after TKA.

## Protocol of administration

There has been no conclusive evidence for the best choice of drugs, usage, and dosage. Boonrionget et al. [33] compared the analgesic efficacy of a single preoperative administration of etoricoxib versus celecoxib. They found that etoricoxib was more effective than celecoxib in controlling postoperative acute pain in patients who underwent arthroscopic anterior cruciate ligament reconstruction. Compared with acetaminophen, oral celecoxib at 200 mg 2 h before operation was a good choice for controlling the postoperative pain in patients who underwent lower extremity surgeries [29]. Administration of selective COX-2 inhibitors both before and after surgery are more effective. A preemptive oral analgesic regimen (200 mg celecoxib, 1 h before surgery) produced a higher threshold, more effective pain relief and lower inflammatory response [19]. Mardani-Kivi et al. [34] advocated that 400 mg celecoxib should be administered 2 h prior to operation to achieve a better pain relief.

## Synergism

Till now, it is difficult to confirm the analgesic effects of preemptive NASIDs, because NASIDs block neither the nociceptive process nor nerve conduction. Currently, NSAIDs combined with opioids are a commonly used protocol. Preemptive analgesia with 3-day administration of celecoxib and low-dose tramadol/APAP is an effective and safe protocol to alleviate postoperative pain [35]. Particularly, preemptive bupivacaine plus morphine usually provides a better pain relief after anterior cruciate ligament reconstruction [36]. Xu et al. [37] found that adding ketorolac is a better method to improve pain relief. Many different protocols have been tried to increase the analgesic effect and decrease the side effects. Future studies should focus on the combined protocols that integrate different drugs and routes (oral, intramuscular, intravenous, epidural, intrathecal, and intra-articular routes).

## Safety of COX-2 inhibitors

No evidence shows negative effects of a short-term adminitration of NSAIDs on the orthopaedic surgical outcomes. However, the anti-infammatory action of NSAIDs may interfere with bone regrowth and osteogenic fusion. In a retrospective trial, Glassman et al. [38] found that administration of ketorolac at a higher daily dose and over a longer period of time resulted in a greater risk of fusion failure. Dimar et al. [39] found that nonsteroidal anti-inflammatory drugs decreased the rate of posterior spinal fusion in rat models. In an animal model, Martin et al. [40] found the rate of spinal fusion was significantly higher compared with the animals who received ketorolac. In a human study, Glassman et al. [41] confirmed that the nonunion rate was five times higher than that of patients treated without ketorolac. However, Reuben et al. [31] argued that there was no difference between the selective NSAIDs and placebo in rat spinal fusion model. Till now, the exact mechanism of NSAIDs working on bone healing remains unclear.

We believe that the selective NSAIDs are effective in controlling the pain after orthopaedic surgeries. However, NSAIDs are contraindicated in patients receiving coronary artery bypass surgery, and those with congestive heart failure, asthma, hypertension, and renal insufficiency [42]. Compared with nonselective NSAIDs, the long-term selective NSAIDs are usually associated with a significantly lower incidence of gastric ulcer and wound bleeding. Nevertheless, the COX-2 inhibitors should be avoided in patients with active bleeding gastric ulcers. Based on the present guideline, proton pump inhibitors should be used if the patients have a history of gastric bleeding. Future studies are necessary to better understand the preemptive analgesic effect of the selective COX-2 inhibitors.

## Conclusions

Preemptive analgesia is an effective technique for pain management after TKA. The selective COX-2 inhibitors decrease the pain by inhibiting central and peripheral sensitization. The selective COX-2 inhibitors are safe analgestic agents without the side effect of wound bleeding.

## Data Availability

Data sharing not applicable to this review as no datasets were generated or analysed during this review.

## Abbreviations

COX: Cycloxygenase enzymes
NMDA: Antagonists N-methyl-D-aspartate receptor antagonists
NSAIDs: Nonsteroidal anti-inflammatory drugs
PGE2: Prostaglandin E2
TKA: Total knee arthroplasty
